# Comment on “Impact of Dose and Sensitivity Heterogeneity on TCP”

**DOI:** 10.1155/2015/682036

**Published:** 2015-02-22

**Authors:** Erik Grusell

**Affiliations:** Department of Medical Physics, Uppsala University Hospital, 75185 Uppsala, Sweden

In [1], the authors give “analytical description and numerical simulations of the influence of macroscopic intercell dose variations and intercell sensitivity variations on the probability of controlling a tumour.” The probability of controlling a tumour is defined as the probability that every tumour cell is killed by the radiation treatment.

The authors use a model for calculating the probability of killing all tumour cells presented earlier by the same authors [2]. However, it was shown in a comment to that article that the model presented there cannot be applied to conventional radiation therapy [3], because, in a clinical megavoltage X-ray radiation field, the kind of variations of absorbed dose assumed in that model does not occur.

In a reply to the comment [4], despite expressly treating “macroscopic intercell dose variations,” the authors refer to microdosimetrical energy deposition variations as motivating their assumption of large and statistically independent variations of absorbed dose over distances of a cell diameter; they write the following.

In this case, one has to keep in mind that microdosimetrical measurements show large variations in specific energy for the same expectation/average value. If we refer to the cell nucleus, the variations could be even higher.

It is of course impossible that “large variations in specific energy” could lead to large variations in the “expectation/average value” (i.e., the absorbed dose) because the latter was supposed to be constant (“the same”).

However, there is no theoretical or experimental support for the occurrence of large and statistically independent variations of absorbed dose over distances of a cell diameter.

For example, as a reasonable model of a clinical radiation field, assume a threedimensional space filled with water for *z* > 0 and empty otherwise. Let photons of, say, 2 MeV impinge on the water surface with their directions parallel to the *z*-axis. Let the dose to a point in the water caused by a single photon be given by *d*_0_(*z*, *r*) where *z* is the depth in the water and *r* is the distance from the point to the line of the initial direction of the photon, $r=\sqrt{{\left(x-x_{0}\right)}^{2}+{\left(y-y_{0}\right)}^{2}}$ where (*x*, *y*, *z*) are the coordinates of the point and (*x*_0_, *y*_0_, 0) is the point where the photon enters the water.

If *N* photons enter the water the dose *d*(*x*, *y*, *z*) to the point (*x*, *y*, *z*) will be given by

(1) $$\begin{array}{c} d\left(x,y,z\right)=\overset{N}{\underset{i=1}{\sum }}d_{0}\left(z,\sqrt{{\left(x-x_{i}\right)}^{2}+{\left(y-y_{i}\right)}^{2}}\right), \end{array}$$

where photon *i* enters the water at (*x*_*i*_, *y*_*i*_, 0).

Assume that on the average *n* photons per unit area hit the water surface and that the photons are distributed at random and that the positions of different photons are statistically independent. The expected value of the dose at a point at the *z*-axis at depth *z* will then be

(2) $$\begin{array}{c} E\left(d\left(z\right)\right)=n\cdot \overset{\infty }{\underset{0}{\int }}d_{0}\left(z,r\right)\cdot 2\pi r\;dr. \end{array}$$

If we use the pencil beam formula from [5] and neglect the scatter component and let the radius of the small volume used to define absorbed dose be *r*_0_ [6], then

(3) d0z,r=Azexp⁡⁡−r0·azr0 for  0≤r≤r0=Azexp⁡⁡−r·azr for  r0<r,

(4) Edzn=Az∫0r0exp⁡−r0·azr0·2πr drEdznW+Az∫r0∞exp⁡−r·az·2π drEdzn≈Az∫r0∞exp⁡−r·az·2π dr.

If *a*(*z*) at 5 cm depth is taken to be *a*(0.05) = 659 m^−1^ [5] and *r*_0_ = 2 · 10^−9^ m [6] and *n* = 2 · 10^15^ m^−2^, corresponding to 2 Gy, then the relative standard deviation caused by the random variation of the photons can be calculated. The result is less than 10^−4^, which is several orders of magnitude smaller than the variations described by Wiklund et al.

Hereby it is shown that the random distribution of the photons has a small effect on the random variations of absorbed dose, so that (4) gives very nearly the absorbed dose for doses used in radiation treatment. As is seen in the calculated dose distributions presented in [5] the dose in the center of the field does not vary much.

However, even if it is hypothetically (and contrary to experimental and theoretical evidence) assumed that dose variations as described by Wiklund et al. should occur, they would not influence the probability of cell survival and hence would not influence the probability of tumour control in the way described by the model of [2].

This is shown as follows. The radiation sensitivity of the cells is described by the function *S*(*d*), the probability of survival of cells of a certain type after a dose *d* of radiation of a certain quality. The function *S*(*d*) is determined experimentally under conditions equivalent to the conditions prevailing under a clinical irradiation, so that if dose variations of the kind assumed in [2] occur in a clinical beam, they are also present when determining *S*(*d*). The dose *d* in the function *S*(*d*) is thus equal to the average dose over a distance of typically 1 mm. It is well known that this average absorbed dose in a clinical photon field varies very little over distances of several mm. See, for example, the calculated and experimental dose distributions in [5].

Therefore, the conclusion remains that the model presented in [2] cannot be applied to radiotherapy, so that the results regarding dose heterogeneity presented in [1] are also not applicable to conventional radiation therapy.

The authors also treat the case of heterogeneous radiation sensitivity of the cells of a tumor, subject to radiation treatment. To that end equation 12 of [1] is used. But that equation is taken from [2], and, as shown in [3] it would only be valid if the radiation sensitivity of a cell during one fraction would be statistically independent of the radiation sensitivity of the same cell during any other fraction and of any other cell during any fraction. This condition is not met for a tumor for the following reasons.

If the variations in radiation sensitivity stem from variations in intrinsic radiation sensitivity, then the sensitivity of the same cell in different fractions will not be independent. Another important cause of variations in sensitivity is variations in oxygenation. In this case cells close to each other will have similar oxygenation, so that their sensitivity variations from this cause are not independent.

Because of this, the results regarding variations in radiation sensitivity using equation 12 are not applicable to radiation treatment.

## References

[1] Wiklund K., Toma-Dasu I., Lind B. K. (2014). Impact of dose and sensitivity heterogeneity on TCP. *Computational and Mathematical Methods in Medicine*.

[2] Wiklund K., Toma-Dasu I., Lind B. K. (2011). The influence of dose heterogeneity on tumour control probability in fractionated radiation therapy. *Physics in Medicine and Biology*.

[3] Grusell E. (2013). Comments on ‘the influence of dose heterogeneity on tumor control probability in fractionated radiation therapy’. *Physics in Medicine and Biology*.

[4] Wiklund K., Toma-Dasu I., Lind B. K. (2013). Reply to the comment on ‘the influence of dose heterogeneity on tumour control probability in fractionated radiation therapy’. *Physics in Medicine and Biology*.

[5] Ahnesjö A., Saxner M., Trepp A. (1992). A pencil beam model for photon dose calculation. *Medical Physics*.

[6] Grusell E. (2015). On the definition of absorbed dose. *Radiation Physics and Chemistry*.

